# Editorial: Insights in infectious agents and disease: 2023/2024

**DOI:** 10.3389/fmicb.2025.1701123

**Published:** 2025-10-16

**Authors:** Axel Cloeckaert

**Affiliations:** INRAE, Université de Tours, UMR, ISP, Nouzilly, France

**Keywords:** viruses, bacteria, fungi, virulence, pathogenesis, pathology, antimicrobial resistance, epidemiology

## 1 Introduction

This third edition of the Research Topic *Insights in infectious agents and disease*, gathered 34 published articles, and provided as in previous editions an overview of recent developments in the field of bacterial, viral, and parasitic infectious diseases. Most publications (*n* = 23) concerned bacterial infectious agents and diseases as in the previous editions.

## 2 Bacterial diseases

Of note for this edition, 10 Review or Systematic Review articles were published. Regarding bacterial pathogenesis, Chakkour et al. provided an overview of *Proteus mirabilis* pathogenicity and virulence with a particular focus on the role of metals, siderophores, iron metal chelating and transporting metallophores, displaying a potential target for therapeutic intervention. Petakh, Oksenych et al. explored the complex interplay between gut microbiome, stress, and leptospirosis, a re-emerging zoonotic disease of significant global health concern. In addition, Petakh, Behzadi et al., in a mini-review, reported also the current treatment options for leptospirosis. Among them, the authors discussed novel treatment options, such as bacteriophages and newly synthesized/natural compounds. Jin reviewed the role of bacterial virulence factors and host elements involved in septic arthritis, together with insights provided from animal models of infection to develop novel therapeutic strategies against this infectious disease. In another Review Article, Li et al. raised an important question regarding the oral-brain axis, in particular if periodontal pathogens can trigger the onset and progression of Alzheimer's disease. In the field of prophylaxis and therapy, Zafar et al. provided an overview of current and emerging approaches for eliminating *Borrelia burgdorferi* responsible for Lyme disease, including currently employed antimicrobial treatment approaches and vaccine candidates. Maurin et al. reviewed non-vaccinal prophylaxis of tularemia, caused by the intracellular pathogen and biological threat agent *Francisella tularensis*. No vaccine is currently available for humans or animals. The authors discuss the currently underdeveloped possibilities for collective prophylaxis which would require a One Health approach to control animal and environmental reservoirs, including the arthropod vectors, to slow current expansion of this disease in a context of climate change. Zahid et al. provided an overview on recent advances in otitis media vaccine developments and animal model systems. Several bacterial pathogens may be involved in otitis media, and therefore the development of multi-species vaccines is required to reduce the global burden of this disease. More largely in the field of vaccine development, Fantoni et al. reviewed the current challenges and improvements in assessing the immunogenicity of bacterial vaccines. In the field of bacterial infection diagnosis, Dixit et al., discussed using a systematic review and meta-analysis approach, the diagnostic accuracy of point-of-care nucleic acid-based isothermal amplification assays for scrub typhus, caused by bacterial pathogens of the genera *Rickettsia* and *Orientia*.

The remaining bacterial infectious diseases-related publications were Original Research articles and concerned mostly genomic studies on specific bacterial pathogens. Ghielmetti et al. presented a new approach for animal tuberculosis surveillance using culture-independent long-read whole-genome sequencing. Related to this study, using culture-independent deep sequencing of DNA, Cooke et al. identified *Mycobacterium bovis* in nasal swabs from communal goats (*Capra hircus*) in rural KwaZula-Natal, South Africa. Regarding wildlife animal tuberculosis, Alba et al. provided genomic insights into a *Mycobacterium pinnipedii* isolate causing tuberculosis in a captive South American sea lion (*Otaria flavescens*) from Italy. Regarding human tuberculosis, Marcon et al., using a comprehensive genomic surveillance approach, identified transmission profiles of extensively drug-resistant *Mycobacterium tuberculosis* cases in Brazil.

Several Original Research publications dealt with enteric pathogens. Kalalah et al. studied the pathogenomes and virulence profiles of representative big six non-O157 serogroup Siga toxin-producing *Escherichia coli*. Zuo et al. provided comparative genomic and antimicrobial resistance profiles of *Salmonella* strains isolated from pork and human sources in Sichuan, China, highlighting the role of pork as a reservoir and transmission source for certain *Salmonella* sequence types. For another important genus/species belonging to the *Enterobacterales* family, Arcari et al. developed a *Providencia stuartii* multilocus sequence typing scheme, useful for subspecies classification and identification. To assess gastrointestinal colonization in experimental models of infection, Wan et al. investigated gut microbiome changes in mouse, Mongolian gerbil, and hamsters following a challenge with another important enteric pathogen, namely *Clostridioides difficile*. In the field of control of enteric pathogens, Shaw et al. studied the interplay between *Salmonella enterica* Typhimurium and the probiotic *Bifidobacterium infantis* during co-infection.

Of importance are also hospital-acquired infections, and Bello-López et al. reported on the emergence of *Acinetobacter pittii* carrying diverse antibiotic resistance genes, which may be responsible for the emergence of this pathogen, together with the high antibiotic selective pressure exerted in the hospital setting.

Regarding zoonotic diseases, Qiu et al. assessed microbiota in organs of the horseflies, constituting an important vector for pathogen transmission, in Northeastern China, and identified an important group of zoonotic pathogens such as *Brucella*. For another important vector borne and more precisely tick-borne disease, Alruwaili et al. reported superior efficacy of combination antibiotic therapy vs. monotherapy in a mouse model of Lyme disease.

More mechanistically in the field of microbial pathogenesis, Chen et al. provided evidence for the role of *Mycoplasma genitalium* adhesion protein in inhibition of human urethral epithelial cells apoptosis via CypA/P13K/AKT/m-TOR-dependent authophagy.

## 3 Viral diseases

One Review article and six Original Research articles concerned human viral infectious diseases of primary importance such as COVID-19 or AIDS. Gurrola et al. provided a review on delivering CRISPR to the HIV-1 reservoirs, as a curative strategy for the inactivation and/or elimination of integrated proviral DNA within host cells. Anderson et al. investigated persistence and risk factors of occult hepatitis B virus infections among antiretroviral therapy-naïve people living with HIV in Botswana.

Regarding COVID-19, da Silva et al. presented a study on genomic surveillance and serological profile of SARS-CoV-2 variants circulating in Macaé and nearby cities, southeastern Brazil. In another study of epidemiological importance, Romano et al. provided phylogenetic and epidemiological insights into centenarians' resilience to COVID-19 by exploring the role of past coronavirus pandemics, from the 19th till 21st century. In the field of therapy, El Khoury and Naim presented experimental evidence that lipid rafts disruption by statins negatively impacts the interaction between SARS-CoV-2 S1 subunit and ACE2 in intestinal epithelial cells, representing a promising therapeutic intervention to treat this viral infection. As another therapeutic approach, Abutbul et al. reported Tumor Treating Fields (TTFields) demonstrate antiviral functions *in vitro*, and safety for application to COVID-19 patients in a pilot clinical study.

For another important viral disease, Ren et al. investigated reactivation of cytomegalovirus and bloodstream infection and its impact on early survival after allogeneic haematopoietic stem cell transplantation.

## 4 Fungal diseases

Two Original Research studies concerned fungal pathogens. Xu et al. reported the clinical isolation, biofilm formation, and pathogenicity analysis of three species of the *Stephanoascus ciferii* complex, namely *Stephanoascus ciferrii, Candida allociferrii*, and *Candida mucifera*. In the field of host-pathogen interaction, Porollo et al. investigated regulatory mechanisms involved in copper sensing and tolerance in *Pneumocystis* species. These fungal pathogens replicate extracellularly in lung alveoli and thrive in the copper-enriched environment of mammalian lungs, hence the importance of studying copper interactions with these pathogenic species.

## 5 Multiple pathogen diseases

Infectious pathology states can also involve diverse multiple pathogenic species at the site of infection, and may be difficult to treat because of their multiple infectious etiology. Fabrizio et al. investigated the efficacy of sodium hypochlorite in overcoming antimicrobial resistance and eradicating biofilms in clinical pathogens from pressure ulcers (PU). The PU isolates analyzed comprised multiple drug resistant bacteria of the ESKAPE group along with fungal *Candida albicans* isolates.

Regarding viral and bacterial co-infections, Maboni et al. provided new insights into the feline respiratory disease complex (FRDC), a highly prevalent syndrome in cats that often result in fatal outcomes. The authors detected a wide range of pathogens involved in this syndrome, of which the most detected were *Mycoplasma*, feline calicivirus (FCV), and felid alphaherpesvirus 1 (FeHV-1). Co-infections of *Mycoplasma* and FCV or *Mycoplasma* and FeHV-1 were found to be important predictors of disease severity.

